# Creation and provision of a question and answer resource for esophageal cancer based on medical professionals’ reports of patients’ and families’ views and preferences

**DOI:** 10.1007/s10388-021-00857-7

**Published:** 2021-06-24

**Authors:** Yasushi Toh, Yoji Inoue, Masayo Hayakawa, Chikako Yamaki, Hiroya Takeuchi, Masaichi Ohira, Hisahiro Matsubara, Yuichiro Doki, Fumihiko Wakao, Tomoko Takayama

**Affiliations:** 1 The Japan Esophageal Society, Tokyo, Japan; 2 Department of Gastroenterological Surgery, National Hospital Organization Kyushu Cancer Center, Fukuoka, Japan; 3 Division of Cancer Information Services, Center for Cancer Control and Information Service, National Cancer Center, Tokyo, Japan; 4 Department of Surgery, Hamamatsu University School of Medicine, Hamamatsu, Shizuoka Japan; 5 Department of Gastroenterological Surgery, Osaka City University School of Medicine, Osaka, Japan; 6 Department of Frontier Surgery, Chiba University Graduate School of Medicine, Chiba, Japan; 7 Division of Gastroenterological Surgery, Department of Surgery, Osaka University Graduate School of Medicine, Osaka, Japan; 8 Center for Cancer Control and Information Services, National Cancer Center, Tokyo, Japan

**Keywords:** Esophageal neoplasms, Academies and institutes, Health communication, Quality of health care, Evidence-based practice

## Abstract

**Background:**

In the rapidly-progressing healthcare environment, it is essential to improve treatment quality through continuous clarification of the needs and concerns of esophageal cancer patients and their families. Effective collaboration between information providers and academic associations could help make such clarified information available.

**Methods:**

We analyzed esophageal cancer patients’ views and preferences (PVPs) using data that were previously obtained from medical staff in Japan. Based on these PVPs, we created a question and answer (Q&A) resource through collaboration with the Cancer Information Service in Japan (CISJ) and the Japan Esophageal Society (JES).

**Results:**

Regarding esophageal cancer, “diet and eating behavior” was the most frequent PVP mentioned by patients and their families, followed by “treatment-related symptoms and adverse effects” and “daily life, recuperation, and survivorship.” These PVPs were noted by a wide variety of medical specialties. By analyzing the PVPs, the CISJ developed 11 proposed questions and sent them to the JES, which then created answers based on evidence and clinical–practice-associated consensus. The resultant Q&A resource was uploaded to the CISJ website with mutual linkage to the JES website.

**Conclusions:**

This study showed the usefulness of collecting esophageal–cancer-related PVPs from medical staff and fostering successful collaboration between a cancer-information provider and an academic association. This arrangement may represent a model case for developing a sustainable system that can satisfactorily respond to PVPs regarding other cancers and/or issues.

## Introduction

Patients with cancer and their families (patients/families) have a wide range of information needs related to treatment plans [1], possible treatment-related adverse effects [2], psychosocial topics [3], and daily activities and values [4, 5]. However, healthcare professionals can be unfamiliar with the novel concerns and problems of patients/families, especially those who lack information or support, referred to as individuals with “unmet needs” [6]. In the rapidly progressing healthcare environment, it is important to identify such needs and improve the quality of information, ensuring that it is available in a prompt and timely manner [7]. Thus, it is necessary to continuously collect comprehensive information regarding the needs and concerns of patients/families and use this information to address these needs.

In our previous study, the authors developed a new method for obtaining a holistic perspective of patients’/families’ views and preferences (PVPs) as expressed to medical staff in various specialties [8]. PVPs were defined as patients’/families’ questions, sense of values, desires, and experiences (including various medical situations) that could provide medical staff with important motivation to improve their clinical practice. Through our research, PVPs regarding five cancer-related topics (colorectal cancer, esophageal cancer, lymphedema, urinary symptoms, and tingling/numbness/pain) were collected from various nationwide cancer-related medical staff [8].

Although high-level scientific evidence regarding medical practice for esophageal cancer is relatively sparse [9, 10], various multimodality treatments have improved the prognosis of patients with esophageal cancer, resulting in increased survivor rates [11]. This means that patients with esophageal cancer and their families need more information concerning the long-term quality of life (QOL) in the post-treatment phase in addition to information such as treatment choices, prognosis and self-care, even before initial treatment begins [12–15]. However, studies concerning esophageal cancer show that the needs of these patients/families are underestimated by physicians [14], and that there is a large interinstitutional difference in the depth and quality of the information used by medical staff to provide consultation and supportive care [15]. To address these problems, it is necessary to develop a system that promptly collects evidence and provides appropriate information suited to the different phases of esophageal cancer in clinical practice. Nevertheless, as a result of insufficiencies regarding budgets and human resources, it is becoming more difficult to develop systems that can respond to requirements that are rapidly increasing in content and volume. Therefore, the construction of novel systems is necessary [8].

In the present study, we analyzed the contents of the esophageal-cancer-related PVPs obtained in our preceding study [8] and attempted to construct a new system for the creation and provision of a question and answer (Q&A) resource that can provide patients/families with appropriate information concerning esophageal cancer. To achieve this, we fostered collaborations between the Cancer Information Service in Japan (CISJ) [16] and the Japan Esophageal Society (JES) [17]; the former is the largest cancer-information provider in Japan, and the latter is a group of Japan-based specialists who periodically publish practical guidelines concerning esophageal cancer. Consequently, we expect that the new model will be adaptable to other kinds of cancer and situations experienced by patients with cancer and will provide accurate information based on the PVPs patients/families commonly present to medical staff.

## Materials and methods

### Collecting patients’ and their families’ views and preferences from medical staff

In our previous study [8], we conducted web-based, anonymous self-administered surveys from July to September 2018 among 904 nationwide cancer-related medical staff of various specialties. These respondents were recruited from 32 hospitals affiliated with the Japanese Association of Clinical Cancer Centers [18] and 434 Cancer Information and Support Centers located in cancer care hospitals designated by the Government of Japan [19]. All hospitals belonging to the former have been contributing to medical improvement of cancer by actively providing cancer information and cancer policy recommendations [18]. The latter which hold many certified cancer counselors are also contributing to provision of cancer information and counseling for cancer patients/families. Thus, the respondents in this study are medical staffs with enough experiences in clinical practice of cancer who are thought to be the best targets in Japan for collecting the PVPs at present [19].

The participants were asked whether they had, within the past year, received questions regarding esophageal cancer. Furthermore, we asked respondents to provide detailed descriptions of the questions they received, if possible [8]. Thus, this questionnaire survey was performed in a simple open-ended manner to collect a wide range of PVPs concerning esophageal cancer through various medical staff, including so far unknown ones, and inductively raise questions to which many patients with esophageal cancer and their families desire answers.

### Views and preferences regarding esophageal cancer and qualitative content analysis

Patients’/families’ statements, as reported by the survey respondents, were qualitatively analyzed using a qualitative content analysis method and were counted after categorization, as described in detail in our previous study [8]. Qualitative content analysis can be defined as ‘a research method for the subjective interpretation of the content of text data through systematic classification process of coding and identifying themes or patterns’ [20]. In this study, we analyzed the statements regarding esophageal cancer by classifying them into eight categories concerning unmet needs and patients’ preferences: (1) symptoms and signs characteristic of esophageal cancer; (2) choice of treatment and second opinion; (3) treatment; (4) treatment-related symptoms and adverse effects; (5) diet and eating behavior; (6) daily life, recuperation, and survivorship; (7) outcome and prognosis; and (8) other [2, 21]. If a statement included two or more meanings or contents, these were counted separately. Analyses were independently performed by two researchers (YI and TT).

According to the ethical guidelines for medical and health research involving human subjects in Japan, approval from an ethical committee was not required for this type of study (reference number 6000-017).

### Developing questions regarding esophageal cancer for the question and answer resource

Based on the esophageal–cancer-related PVPs identified through content analysis, questions for which patients/families strongly desire answers but could be difficult for medical staff to answer were developed by the CISJ. Both the CISJ (through its website and various booklets [16]) and the JES (through published guidelines and its website [17]) provide extensive information regarding esophageal cancer “treatment.” Therefore, when creating the Q&A resource we excluded “treatment” from the target categories.

## Results

### Views and preferences regarding esophageal cancer and the qualitative content analysis

Of the 904 medical staff who responded to our survey [8], PVPs regarding esophageal cancer were obtained from 333 participants, comprised of a wide variety of medical specialties, including physicians, nurses, and pharmacists. Backgrounds and characteristics of these respondents are shown in Table 1. Approximately 70% of 333 respondents had clinical experiences of more than 10 years. They provided a total of 627 PVPs, which were classified into the eight categories mentioned above (Table 2). Among these PVPs, “diet and eating behavior” represented the largest category (224; 35.7%), with “anxiety” being the most common topic in this category. PVPs concerning “treatment-related symptoms and adverse effects,” especially surgery-related effects, were the second-most common (99; 15.8%). In addition, many PVPs relating to other esophageal-cancer topics were observed, most notably regarding “daily life, recuperation, and survivorship”.

**Table 1:** Respondents' demographic and clinical characteristics

Variables	*n* (%)
Sex	
Male	82 (24.6)
Female	250 (75.1)
Unknown	1 (0.3)
Age	
20–29	40 (12.0)
30–39	93 (27.9)
40–49	110 (33.0)
50–59	73 (21.9)
≥ 60	16 (4.8)
Unknown	1 (0.3)
Length of clinical experience	
< 3 years	11 (3.3)
3–5 years	30 (9.0)
5–10 years	56 (16.8)
10–20 years	105 (31.5)
> 20 years	125 (37.5)
Unknown	6 (1.8)
Medical speciality	
Physician	59 (17.7)
Pharmacist	39 (11.7)
Nurse	76 (22.8)
Physical/occupational/speech therapist, radiation/clinical laboratory technologist	61 (18.3)
Dietitian	25 (7.5)
Clinical psychologist, social worker	23 (6.9)
Cancer counsellor	38 (11.4)
Medical clerk	12 (3.6)
Total	333 (100)

**Table 2:** Categorization of patients' views and preferences related to esophageal cancer

Category	*n*
Symptoms	34
Esophageal–cancer-specific symptoms	25
Pain	9
Selection of treatment	48
Selection of treatment	44
Second opinion	4
Treatment	78
Standard treatment (surgical)	29
Standard treatment (non-surgical)	24
Non-standard treatment	8
Other	17
Treatment-related symptoms and adverse effects	99
Stents and adverse effects	6
Surgery and adverse effects	64
Chemotherapy and adverse effects	12
Radiotherapy and adverse effects	12
Other and associated adverse effects	5
Diet and eating behaviors	224
Anxiety	81
Difficulty	53
Nutrition	69
Alcohol intake	7
Other	14
Daily life, recuperation, and survivorship	83
Body weight	10
Vocalization	22
Physical therapy	8
Other	28
Work, rehabilitation to work	15
Outcome and prognosis	19
Recurrence, metastasis,	3
Survival rates, prognosis	16
Other	17
Other	17
No answer	25
No answer	25
Total	627

The number and proportion of PVPs from each category encountered by each medical specialty are shown in Table 3. “Diet and eating behavior” (representing 37.2% of the total PVPs that could be categorized) was the most common category encountered by dietitians (70.4%), clinical psychologists and social workers (54.2%), nurses (35.5%), and cancer counselors (35.5%). Physicians encountered a wide range of PVPs relating to categories such as “treatment,” “treatment-related symptoms and adverse effects,” and “daily life, recuperation, and survivorship.” This tendency was also observed among nurses and pharmacists.

**Table 3:** Medical professionals' experience of patients' views and preferences for each category, listed in terms of their medical speciality

	Number of PVPs received	Symptoms	Selection of treatment	Treatment	Treatment-related symptoms and adverse effects	Diet and eating behaviors	Daily life, recuperation, and survivorship	Outcome, prognosis	Other	Total
Medical profession		*n* (%)	*n* (%)	*n* (%)	*n* (%)	*n* (%)	*n* (%)	*n* (%)	*n* (%)	*n* (%)
Physician	59	5 (6.4)	9 (11.5)	10 (12.8)	15 (19.2)	15 (19.2)	13 (16.7)	4 (5.1)	7 (9.0)	78 (100)
Pharmacist	39	3 (7.0)	2 (4.7)	8 (18.6)	10 (23.3)	14 (32.6)	3 (7.0)	2 (4.7)	1 (2.3)	43 (100)
Nurse	76	9 (4.9)	14 (7.7)	32 (17.5)	30 (16.4)	65 (35.5)	23 (12.6)	7 (3.8)	3 (1.6)	183 (100)
Physical/occupational/speech therapist, radiation/clinical laboratory technologist	61	8 (13.1)	0 (0.0)	3 (4.9)	10 (16.4)	17 (27.9)	17 (27.9)	3 (4.9)	3 (4.9)	61 (100)
Dietitian	25	3 (4.2)	0 (0.0)	2 (2.8)	11 (15.5)	50 (70.4)	5 (7.0)	0 (0.0)	0 (0.0)	71 (100)
Clinical psychologist, social worker	23	0 (0.0)	1 (4.2)	3 (12.5)	1 (4.2)	13 (54.2)	5 (20.8)	1 (4.2)	0 (0.0)	24 (100)
Cancer counselor	38	6 (4.4)	21 (15.2)	20 (14.5)	21 (15.2)	49 (35.5)	17 (12.3)	2 (1.5)	2 (1.5)	138 (100)
Medical clerk	12	0 (0.0)	1 (25.0)	0 (0.0)	1 (25.0)	1 (25.0)	0 (0.0)	0 (0.0)	1 (25.0)	4 (100)
Total	333	34 (5.6)	48 (8.0)	78 (13.0)	99 (16.4)	224 (37.2)	83 (13.8)	19 (3.2)	17 (2.8)	602

Of the total answers, 25 were excluded due to a lack of PVPs

*PVPs* patients’ views and preferences

### Creation and provision of a question and answer resource regarding esophageal cancer through collaboration between the CIS and JES

**Fig. 1:**
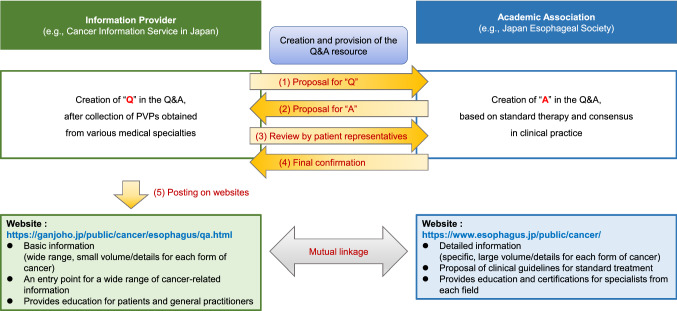
Collaboration model for an information provider and an academic association

A total of 11 questions were developed, covering six categories of esophageal-cancer-related PVPs (Table 4). Four of the 11 questions concerned “diet and eating behavior,” which was the most frequently expressed PVP; two concerned “treatment-related symptoms and adverse effects” and “daily life, recuperation, and survivorship.” As specialist cooperation was necessary to create specific answers for each question, the CISJ provided the JES with the proposed questions. The JES then created answers for these questions by incorporating existing evidence and consensus in clinical practice. Moreover, the resultant Q&A resource was reviewed by the CISJ Patient-Public Panel [22] in terms of readability and understandability from the patient perspective. After a final revision, the Q&A resource was then uploaded to the CISJ website [16] (Fig. 1).

**Table 4:** Questions and answer resource for esophageal cancer, based on the patients' views and preferences

Category	Q#	Question
Symptoms	Q1	What kind of symptoms appear when the esophageal cancer advances?
Selection of treatment and second opinion	Q2	How do I choose between treatment, surgery, chemotherapy, and radiotherapy?
Treatment-related symptoms and adverse effects	Q3	To what degree will each treatment affect my life?
	Q4	What kind of symptoms will appear after treatment?
Daily life, recuperation, and survivorship	Q5	How long after treatment can I return to work or ordinary life?
	Q6	Will vocalization become difficult after treatment?
Diet and eating behaviors	Q7	Will discomforts such as swallowing difficulty or passage disturbance be relieved after radiotherapy?
	Q8	What kind of diets are beneficial or harmful after treatment?
	Q9	What do you recommend I do if I experience difficulty dieting?
	Q10	Is alcohol intake or smoking permitted after treatment?
Prognosis	Q11	How can I ask a physician about my future clinical course?

Answers are available on the website of the Center for Cancer Control and Information Services, the National Cancer Center (NCC-CIS), in Japanese: https://ganjoho.jp/public/cancer/esophagus/qa.html

## Discussion

In the present study, we presented a successful model for collaboration between information providers (such as the CISJ) and academic associations (such as the JES) to achieve the proper and timely provision of PVP-based Q&A resources for patients/families.

This report clarified that patients with esophageal cancer and their families strongly desire information concerning “diet and eating behavior.” Surgery, a mainstay of treatment for esophageal cancer for advanced stages of the illness [10, 23] imposes enormous stress on patients and is usually accompanied by the loss of the esophagus, patients’ postoperative daily lives are extensively influenced for a long time [15, 24]. For example, Boshir et al. showed, in a cohort study of the long-term health-related QOL (HR-QOL) of disease-free survivors of esophageal cancer, that body-weight loss and other gastrointestinal symptoms (except dysphagia) persist up to 20 years after surgery [24]. Furthermore, a review concerning supportive care for patients with esophageal cancer indicated that such patients’ long-term HR-QOL is markedly influenced by changes in dietary habits and social identity, as well as psychological distress [15]. Thus, it is important that medical staff develop problem-awareness concerning the most frequently expressed category of esophageal–cancer-related PVPs, “diet and eating behavior,” and attempt to provide timely and appropriate information regarding this topic. Such attitudes may help foster an environment where patients/families feel free to ask questions and voice concerns to medical staff, and easily gain the information they desire.

It is reported that partnerships between the healthcare system and academia are important for improving health service delivery, but that there is limited guidance for either healthcare organizations or academic researchers regarding how to select, build, and manage effective partnerships [25, 26]. In this paper, we reported the successful development of a system in which the CISJ and JES collaborated to create and provide information concerning esophageal cancer in the form of a Q&A resource that was based on PVPs collected from medical staff. For many PVPs, such as “daily life, recuperation, and survivorship” and “diet and eating behavior,” there is a lack of information available; the response, therefore, usually depends on the experience and knowledge of the individual who is asked; this often leads to interinstitutional differences [27]. To solve this problem, precise information should be provided in an easily understandable form to all medical staff as well as patients/families. Our previous study showed that one-third of medical staff do not have enough resources to answer PVPs regarding esophageal cancer [8]. Thus, the system reported in the present research might be beneficial for both medical staff and patients with esophageal cancer and their families. Furthermore, the collection of PVPs by medical staff regarding specific topics might represent an effective substitute for large-scale opinion research of patients/families or general citizens. It would also be more economical in terms of budget and time, despite the indirect approach involved. Meanwhile, as academic associations and groups of specialists usually have difficulty in determining the PVPs of targeted patients [28, 29], collaboration with an information provider (e.g., CISJ) can help such associations identify PVPs of interest and help develop detailed answers or information for patients/families. This kind of collaboration system could represent a model case for precise and prompt information provision concerning both other cancers and issues, for which there is a limited amount of evidence-based information.

In addition, a mutual linkage of the CISJ and JES websites has been developed, in addition to the creation and provision of the Q&A resource for esophageal cancer (Fig. 1). In arbitrary terms, the CISJ website provides patients/families with broad, but rather limited, information regarding various cancers, while that of the JES concentrates on diagnosis and treatment of esophageal cancer, with relatively little information regarding other areas such as “daily life, recuperation, and survivorship,” and “diet and eating behavior.” By linking to each other, patients/families can obtain the information they desire by visiting the CISJ site and then the JES site, or vice versa, depending on which they access first. The Q&A resource, which is informative and simple to understand, contributes to this arrangement by representing a starting point that can help patients/families make more precise decisions and source more detailed information from these websites.

There are some limitations to this study. The first is the indirect collection of PVPs. Some PVPs may have been emphasized by the medical staff as a result of their unique concerns and individual medical experiences, while other PVPs that were less related to their medical specialties or interests may have been neglected. Second, in the content analysis of the PVPs concerning esophageal cancer, detailed information regarding patients’ conditions, such as stage, current treatment status, and the clinical course was not known. More detailed data may have resulted in different results being observed, depending on individual conditions [30]. The third limitation is that it may be difficult to develop a collaborative system between other information providers and academic associations because there is usually a limited relationship between them.

In conclusion, we presented the usefulness of collecting cancer-related PVPs through medical staff and fostering successful collaboration between cancer-information providers and academic associations using esophageal cancer as an example. Further sustainable systems regarding other cancers can be designed using collection of PVP through medical staff and interaction between providers and associations in mutually beneficial relationships can be designed to provide patients/families with precise and satisfactory information. Further research is needed to clarify whether developing Q&A regarding PVPs frequently encountered by medical staff will lead to promoting studies that establish evidence or encourage changes for those who create and provide clinical practice guidelines. Furthermore, additional researches can be conducted on whether patients’/families’ access to the Q&A will accelerate their understanding of information regarding the disease and interactions with medical staff.

## Data Availability

The datasets analyzed during the current study are not publicly available as they are currently being used in the preparation of manuscript but are available from the corresponding author on reasonable request.
